# Implication of Voltage-Gated Potassium Channels in Neoplastic Cell Proliferation

**DOI:** 10.3390/cancers11030287

**Published:** 2019-03-01

**Authors:** Clara Serrano-Novillo, Jesusa Capera, Magalí Colomer-Molera, Enric Condom, Joan Carles Ferreres, Antonio Felipe

**Affiliations:** 1 Molecular Physiology Laboratory, Departament de Bioquímica i Biomedicina Molecular, Institut de Biomedicina (IBUB), Universitat de Barcelona, Avda. Diagonal 643, E-08028 Barcelona, Spain; clara.serrano.n@gmail.com (C.S.-N.); 11jesusa@gmail.com (J.C.); magali@colomer.cat (M.C.-M.); 2 Departament de Patologia i Terapèutica Experimental, Hospital Universitari de Bellvitge-IDIBELL, L’Hospitalet de Llobregat, 08907 Barcelona, Spain; ecm@bellvitgehospital.cat; 3 Departament de Ciències Morfològiques, Parc Taulí Hospital Universitari, Universitat Autònoma de Barcelona, 08208 Barcelona, Spain; joancfp@gmail.com

**Keywords:** K^+^ channels, cancer, tumor progression, cell cycle, proliferation

## Abstract

Voltage-gated potassium channels (Kv) are the largest group of ion channels. Kv are involved in controlling the resting potential and action potential duration in the heart and brain. Additionally, these proteins participate in cell cycle progression as well as in several other important features in mammalian cell physiology, such as activation, differentiation, apoptosis, and cell volume control. Therefore, Kv remarkably participate in the cell function by balancing responses. The implication of Kv in physiological and pathophysiological cell growth is the subject of study, as Kv are proposed as therapeutic targets for tumor regression. Though it is widely accepted that Kv channels control proliferation by allowing cell cycle progression, their role is controversial. Kv expression is altered in many cancers, and their participation, as well as their use as tumor markers, is worthy of effort. There is an ever-growing list of Kv that remodel during tumorigenesis. This review focuses on the actual knowledge of Kv channel expression and their relationship with neoplastic proliferation. In this work, we provide an update of what is currently known about these proteins, thereby paving the way for a more precise understanding of the participation of Kv during cancer development.

## 1. Potassium Channels: Classification and Function

Ion channels are transmembrane proteins that form aqueous pores and drive the selective flow of ions, participating in the electrochemical gradient across the cell membrane. They are fundamental for excitable cells but are also involved in cell functions, such as proliferation, migration, cell volume, and specific processes such as insulin release or muscular contractibility [1]. Their participation in such highly diverse phenomena highlights a crucial biological relevance. Thus, mutations and alterations of the normal function of these proteins trigger alterations, called channelopathies, in cardiovascular and nervous systems as well as autoimmune and metabolic diseases. [2,3].

The British Pharmacological Society (BPS) and the International Union of Basic and Clinical Pharmacology (IUPHAR) (http://www.guidetopharmacology.org/) classify ion channels as (i) voltage-gated ion channels, (ii) ligand-gated ion channels, or (iii) channels using other gating mechanisms, including aquaporins, chloride channels, and store-operated calcium channels. Following these criteria, 141 members are included in the voltage-gated ion channel superfamily, making it one of the largest groups of signal transduction proteins [4,5].

Potassium-selective channels (K^+^ channels) are the largest and most diverse group of voltage-gated ion channels expressed in both excitable and nonexcitable cells. K^+^ channels include four of the 11 families of the voltage-gated ion channel superfamily: (i) Voltage-gated K^+^ channels (Kv); (ii) Ca^2+^– and Na^+^– activated K^+^ channels (K_Ca_, K_Na_); (iii) inwardly rectifying K^+^ channels (Kir); and (iv) two-pore domain K^+^ channels (K_2P_). In addition to pore-forming subunits of the K^+^ channels (α subunits), they associate with several auxiliary subunits (β subunits), which increases the diversity of roles and implications of channels in health and disease. The diversity of α and β subunits—added to a wide range of pre- and posttranslational processes controlling protein expression, traffic, assembly, and/or function—configure the myriad of pathological dysfunctions, including cancer [3,6].

## 2. Potassium Channels in Cancer

Cancer is a multifactorial process. Cells acquire an atypical phenotype caused by genetic and/or aberrant protein expression. Cells proliferate massively and are mostly resistant to apoptosis. During tumorigenesis, several phenotypical alterations occur. Altered ion channel expression leads to modifications that could favor tumor progression [7]. 

Cancer cell studies should include the tumoral microenvironment, which contains mesenchymal, endothelial, and immune cells, as well as extracellular matrix proteins and soluble factors. This microenvironment plays an important role in tumor progression being responsible for cell-cell interactions, as well as for cell-matrix signals. Sometimes, the relationship between cancer and the immune system response against the tumor stimulates a favorable environment for tumor progression. Some tumor cells, escaping from the attack of leukocytes, become resistant tumor cells. Thus, the tumor loses immunogenicity and stimulates the production of antiapoptotic cells, generating an immunosuppressing system in the microenvironment that ends with the immune system failure to control tumor growth [8,9]. 

**Figure 1:**
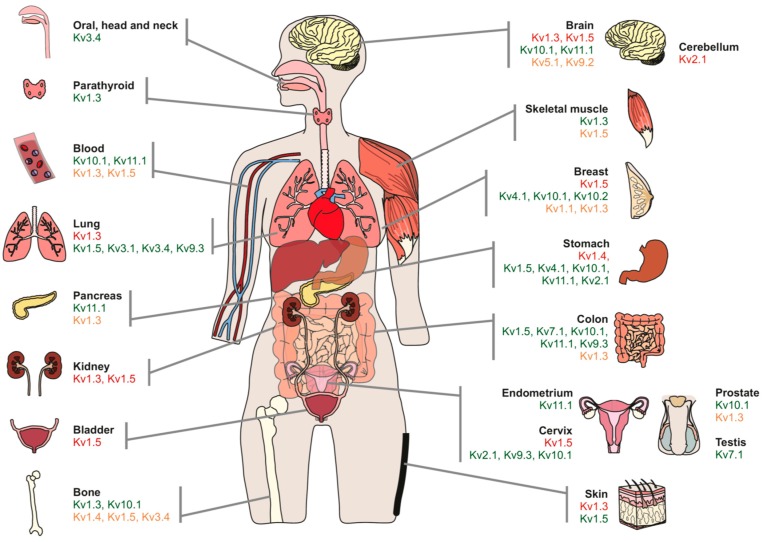
Remodeling of voltage-gated K^+^ channels (Kv) channel expression in human cancers. Schematic representation of the human body highlights the Kv distribution in tumors. Many studies document changes in Kv channel expression (see text for details). Colors represent differential levels of expression: Red, down-regulation; green, up-regulation; orange, altered expression (evidence claim opposite effects in the Kv channel abundance).

Evidence has documented a close relationship between ion channels and cancer, supporting a pivotal role for K^+^ channels in cancer therapy. The specific point where ion channels are involved in tumorigenesis remains unclear, as does how K^+^ channels remodel under neoplastic cell proliferation (Figure 1). However, a pharmacological K^+^ channels blockade impairs proliferation [7,10]. Highly proliferative cells are more depolarized than differentiated or quiescent cells. However, transient hyperpolarization is needed for progression during the first stages of the cell cycle (G1→S). Therefore, a change in the membrane potential must occur for cell cycle progression, as well as during cell migration and adhesion and cytokine production against the tumor. These phenomena require the participation of ion channels, including voltage-gated potassium channels (Kv). Evidence has suggested that Kv control a check point around the initial stages of the cycle, fitting with the change in the membrane potential, cell volume control, and other ion channel regulation, such as Ca^2+^-dependent ones. However, it is important to highlight that, although Kv are involved in proliferation, only few trigger clear oncogenic effects [11,12,13,14,15,16]. 

K^+^ channels are potential molecular targets for anticancer therapies. Thus, K^+^ channels blockers and anti-K^+^ channels antibodies are used. Animal toxins present high affinity for the channels, but targeting K^+^ channels during cancer without harmful side effects, such as cardiac arrhythmias, is a task worthy of effort [17,18]. For example, clofazimine promotes neoplastic B-cell death by inhibiting Kv1.3 in chronic lymphocytic leukemia [19]. Kv1.1 blockers from scorpion venoms, such as KAaH1 and KAaH2, inhibit cell migration and adhesion in colon adenocarcinoma, breast cancer, and glioblastoma, but affect neither cell cycle progression nor apoptosis [20]. The effect of the treatment is associated with the channel abundance. Thus, the tricyclic antidepressant imipramine, an antidepressant Kv10.1 antagonist, improves the survival rate better in patients with moderate Kv10.1 expression in brain cancers [21]. Finally, Kv11.1 participate in the P13K/Akt-dependent pathway that induces hypoxia-inducible factors (HIF) and vascular endothelial growth factor (VEGF) to promote gastric tumor progression. Kv11.1 blockade inhibits cell growth, angiogenesis, and metastasis [22]. 

## 3. Kv Channels and Cancer

Kv exhibit specific physiological and pharmacological properties, and cells could express a variable repertoire of channels. According to their functional properties, Kv are grouped into four families [1,5]. In this review, we will structure the information considering this functional classification. 

### 3.1. Delayed Rectifier Channels (I_DR_) 

**Figure 2:**
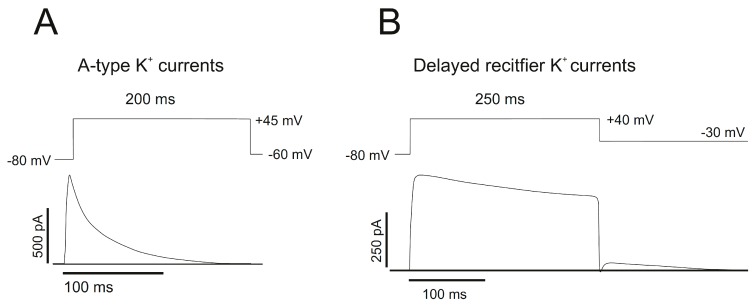
Representative outward A-type and delayed rectifier K^+^ currents. Voltage-clamp records of Kv1.4 and Kv1.5 currents expressed in mouse L-cells. Results shown are traces obtained for depolarization test potentials as indicated. (**A**) Kv1.4 currents rapidly inactivated during maintained depolarization. (**B**) Kv1.5 displays fast activation and slow and only partial inactivation.

Delayed rectifier channels exhibit a delay before activation (Figure 2). They generate an outward current of K^+^ following membrane depolarization triggered by an influx of Na^+^ ions inside the cell. To counteract this cation influx, I_DR_ channels allow the exit of K^+^ ions from the cell. Therefore, the membrane repolarizes, shortening the duration of the nerve impulse. This is crucial in excitable cells such as neurons or muscle cells, but their presence is ubiquitous in the human body. This group includes members of the Shaker-related family (Kv1.1–Kv1.3, Kv1.5–Kv1.8), the Shab-related family (Kv2), some Shaw-related members (Kv3.1, Kv3.2), the Kv7 group and Kv10.1, from the ether-à-go-go (EAG) family. 

Some I_DR_ participate in neoplastic phenomena. Shaker-related members, such as Kv1.3 and Kv1.5, play an important role in cell proliferation (e.g., in macrophage; astrocytes; and muscular, vascular, and skeletal cells). These channels remodel their expression during both physiological and neoplastic cell growth. In fact, evidence has demonstrated altered expression in several types of tumors and cancer cell lines [23]. Kv1.3 and Kv1.5 are the major Kv channels in leucocytes, and, because Kv control crucial functions such as cell proliferation, activation, migration, or apoptosis, it is not surprising that blood cancers remodel these channels. However, their pattern is not always similar. For instance, Kv1.5 is differentially expressed in various tumors. Furthermore, Kv1.5 is inversely correlated with tumor aggressiveness in non-Hodgkin’s lymphomas [24], whereas Kv1.3 is decreased in lymphoma and leukemia samples but is not always related to tumor malignancy [25,26]. In fact, Kv1.3 could function as a tumor suppressor in blood cancers by a mechanism that implies apoptosis [27,28]. 

Kv1.3 expression is well documented in solid tumors. Both pro- and anti-proliferative properties have been assigned to this channel, depending on the tissue and the stage and degree of malignancy of the tumor. Kv1.3 is differentially remodeled in breast, colon, lung, glioma, muscle, brain, or prostate cancers [23,26]. Its role in tumor progression is not clear, and different implications are described depending on the cancer. Sometimes Kv1.3 expression is aberrant and related to proliferation and apoptosis [19,29,30,31], whereas only cell migration and adhesion are altered in others [20]. 

Similarly, some examples inversely correlate Kv1.5 and malignancy, linking the channel with apoptosis and impairing cancer progression [23,32]. However, Kv1.5 is overexpressed in some malignant and aggressive neoplasia, such as gastric, bone or colon cancers, where it participates in tumor proliferation and calcium homeostasis [25,33,34]. Furthermore, Kv1.5 is overexpressed in muscle sarcoma and is related to tumor malignancy [35,36]. By contrast, and similarly to lymphomas, a Kv1.5 abundance is inversely correlated with the degree of malignancy in gliomas [37]. Moreover, the methylation of Kv1.3 [38] and Kv1.5 [39,40] promoters silences channel expression in some neoplastic phenotypes, which supports their roles as tumor suppressors. Both Kv1.3 and Kv1.5 are upregulated during the initial phases of the cell cycle, thus promoting cell cycle progression. Therefore, both channels undergo cell cycle-dependent regulation; however, the molecular mechanisms remain poorly understood [41].

Mitochondria, playing a pivotal role in cell metabolism, participate in apoptosis. Mitochondria contribute to reactive oxygen species (ROS) production and the onset of signaling pathways [42]. Bcl-2 family members, such as Bax, inhibit mitochondrial channels, such as mitoKv1.3 and K_Ca_3.1, downstream of pro-apoptotic signals to promote cell survival [27]. Other mitochondrial K^+^ channels, such as KCa2.x or TASK-3, have been related to cell death, suggesting a potential link between K^+^ channel modulation and intrinsic apoptosis [42,43]. Cancer therapies targeting mito-channels, such as mitoKv1.3, selectively reduce tumor cells and control cancer development and progression in mouse models of pancreatic ductal adenocarcinomas (PDAC) and melanoma [43,44,45]. Considering the above, ion channels, such as Kv1.3 and Kv1.5, should be considered multifunctional proteins; therefore, assuming a single role is a misinterpretation. 

Kv1.1 has been documented in breast cancer and, similar to Kv1.3, plays different roles. Kv1.1 functions as a tumor suppressor when it changes its cell location, affecting cellular senescence and transformation [46]. On the other hand, breast cancer cell lines show Kv1.1 overexpression and, similar to other Kv channels, implicates it in cell migration and tumor development [20]. Kv2.1 is also altered in several cancers, such as gastric [33], medulloblastoma [47], or endometrial cancer [48]. As we will explain later, Kv2.1 exhibits a cell cycle-dependent subcellular distribution, concentrating in raft-like lipid domains during M phase [49]. Thus, anti-neoplastic treatments targeting lipid rafts affect Kv2.1 function [50]. 

Some members of the KCNQ (Kv7) family are also related to cell proliferation and cancer. Kv7.1 remodels in some tumors, and channel inhibition reduces cell proliferation. Kv7.1 is increased in colon cancer as well as in seminoma and germinal cell line tumors [51,52]. Additionally, Kv7.5, which contributes to the vascular smooth muscle tone, participates at the G1/S phase transition during cell cycle progression in myoblasts [53]. 

Ether-a-go-go potassium channels (hEAG/Kv10.1) are characterized by the correlation between the speed of activation and membrane potential before the stimulus. These channels undergo cell cycle regulation. In fact, Kv10.1 was the first voltage-gated channel related to oncogenesis. Many cell and tumor models document the relationship between Kv10.1 expression and tumor growth [54,55]. Kv10.1 expression is mostly restricted to the central nervous system under healthy conditions. However, noticeable Kv10.1 levels are detected in clinical tumors from several different origins, including neuroblastomas [56], glioblastomas and derived brain metastasis [21], breast cancer [57,58], colon and gastric cancers [59,60], or osteosarcomas [61,62]. This evidence supports Kv10.1 as a potential marker for several cancers, such as cervical and colon cancer. Aberrant expression of Kv10.1 correlates with a malignant phenotype and a poor survival rate, probably because the channel provides a good environment for tumor development. Kv10.1 stimulates vascularization and certain resistance to hypoxia, both of which are an advantage for the survival of tumor cells against an immune attack. Kv10.1 is also related to cytoskeletal regulation, which may be associated with proliferation, cell adhesion, and metastasis [58,63,64]. 

### 3.2. A-Type Channels (I_A_) 

I_A_ channels generate a transient-outward K^+^ current with little delay after depolarization (Figure 2). Characterized by rapid inactivation, these channels open when depolarization occurs after hyperpolarization, and they increase the interval between action potentials. Thus, I_A_ help neuronal repetitive firing. This group includes members of the Shaker (Kv1.4), Shaw-related (Kv3.3, Kv3.4), and Shal-related (Kv4) families. 

Kv1.4 expression is impaired in gastric cancer because of hypermethylation of the promoter, resulting in a loss of channel expression [65]. Kv3.4 is present in oral carcinomas, head and neck cancers, and leukoplakia samples, together with altered ROS production patterns, hypoxia-related tumor processes, and cell cycle arrest-mediated control of proliferation [66,67,68,69,70]. Kv1.4, as well as Kv3.4, are also present in bone cancer and show changes in expression and in function when related to pain. However, the role of the channels in proliferation or tumorigenesis is not clear [71]. Cell migration and invasion are altered in aggressive lung adenocarcinoma cell lines overexpressing Kv3.4 [69]. On the other hand, the aberrant expression of Kv4.1 is documented in gastric and mammary cancers. In this vein, Kv4.1 inhibition halts cancer proliferation by arresting cells at the G1/S transition of the cell cycle [72,73]. 

### 3.3. Modifier/Silencer Subunits 

Several groups have similar sequences and structures to those of some Kv families but are not functional in homotetrameric compositions. Instead, they mostly heterotetramerize with members of the Kv2 family, modulating their activity. This group includes the Kv5, Kv6, Kv8, and Kv9 families. These channels present a restricted tissue expression, indicating a tissue-specific function for the heterotetrameric channels. Scarce information is related to these channels regarding cancer. However, their expression is impaired in some cancer cell lines, and evidence suggests that they are involved in cell proliferation, both acting as nonconduction proteins or associated with Kv2.1 [74]. For example, Kv9.3 and Kv2.1 could be major components of Kv channels in cervical adenocarcinoma cells, linking with cell cycle regulation [48]. By contrast, in colon and lung adenocarcinomas, Kv9.3 overexpression by itself could also be related to tumor progression. Silencing Kv9.3 but not Kv2.1 in these cancer cell lines inhibits cell proliferation, causing G0/G1 cell cycle arrest [75].

### 3.4. Others 

Some channels cannot be grouped into any of the abovementioned categories according to their properties. For example, Kv10.2 is sometimes defined as a noninactivating outward-rectifying potassium channel. In addition, Kv11.1, a member the hERG family, is a voltage-gated potassium channel with inwardly rectifying properties. Finally, K_Ca_3.1 channels are activated in response to voltage and Ca^2+^ changes. 

Kv11.1 is mainly expressed in the heart but shows certain ubiquitous expression in the remaining healthy human tissues. This channel is present in several tumors from multiple origins, such as gastric, colorectal, pancreatic, neuroblastoma, leukemia, or endometrial cancers [22,76,77,78,79,80]. In these neoplasias, Kv11.1 causes resting-potential variations along different stages of the cell cycle in tumor cells. Evidence correlates Kv11.1 with malignancy and prognosis of the cancer [79,81,82]. During cancer progression, Kv11.1 participates with the stimulation of angiogenesis and the recruitment of cytokines or growth factors. Thus, this channel functions during differentiation, cell migration, invasiveness, and proliferation, which can be an advantage for cancer cells [83,84,85].

## 4. Regulation of Cell Cycle Progression by Kv Channels

**Figure 3:**
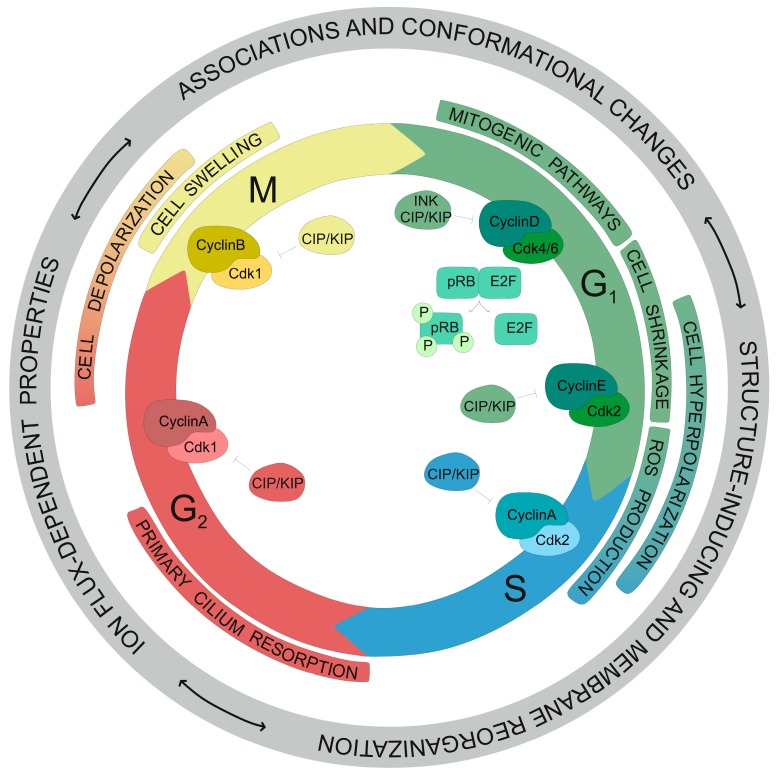
Participation of potassium channels on the control of the cell cycle progression. Kv channels participate during the cell cycle in a series of events that control the progression. Events indicated from outside to inside circles. Outer grey circle: Physical and biochemical properties of ion channels affecting cell cycle progression: (i) Ion flux-dependent properties due to K^+^ conduction; (ii) Kv conformational changes may associate with other down-stream signalling partners; and (iii) Kv channels can also induce membrane reorganization phenomena and promote the formation of subcellular structures. These connected events, related to no specific phase, contribute to the regulation of Ca^2+^ oscillations leading to cell cycle progression. Inner colored circles: Events regulated by Kv channels in specific phases of the cell cycle. Colors correspond to sequential phases of the cycle. Color gradients represent transitions between phases. Kv channels regulate membrane potential, cell volume, mitogen-dependent signal transduction pathways, and other processes involved in cell cycle progression, such as the primary cilium resorption and mitochondrial ROS production. Cell cycle representation: M (yellow) and S (blue) phases of the cell cycle are separated by G1 (green) and G2 (red) gap phases. Several CDK-Cyclin complexes and CDK-inhibitors regulate transitions between phases. In the inner circle, colored complexes are active at specific stages of the cell cycle.

Several redundant and independent mechanisms finely control proliferation and cell cycle progression in all cell types (Figure 3). Such a set of molecular events operates through a checkpoint system which guarantees the initiation of an event only after the successful completion of the preceding step. This checkpoint system organizes the cell cycle in different phases named G1 (gap 1), S (DNA synthesis), G2 (gap 2), and M (mitosis). After mitosis, the cell either can move on to a new G1 phase or enter into a quiescent state. This latter state is representative of end-differentiated cells, which will last for the rest of their lifetime. Transition between phases is regulated by the cyclic activation/inactivation of cyclin-dependent kinases (CDKs) by cyclins and CDK inhibitors (CKIs), respectively. K^+^ channels can control the upstream biochemical events leading to cell cycle progression by the regulation of biophysical properties such as the membrane potential and cell volume, in addition to mechanisms involving protein-protein interactions—all of which converge in tight regulation of Ca^2+^ oscillations.

The observation that the membrane potential is not constant during the cell cycle dates back to the last century. However, at that time, the causal relationship was not clear [86,87]. Since then, evidence has suggested the bioelectric control of the cell cycle [88]. An increase in K^+^ permeability hyperpolarizes the cell at the end of the G1 phase. By contrast, depolarization is found at the G2/M border. These changes in the membrane potential are gradual rather than instantaneous and have been proven to be essential for the proliferation of many cell types. Thus, inhibition of K^+^ channels activity produces cell cycle arrest, typically by hindering G1/S transition [89,90]. Interestingly, although cell cycle-dependent fluctuations in the membrane potential are observed under both physiological and pathophysiological conditions, cells with a high proliferative phenotype tend to be more depolarized at each step than their normal analogs. Therefore, malignant cancerous cells show a depolarized phenotype, and depolarization itself can induce cancerous transformation. Indeed, depolarization has been suggested as a hallmark of cancer [90]. Growing evidence is unravelling a complex scenario where not only the type of current but also the molecular identity of the potassium channel is important to regulate a function that, in most cases, is time and place dependent.

For instance, as abovementioned, Kv1.5 but not Kv1.3 activity is important for myoblast proliferation; however, both channels are transcriptionally upregulated during G1 phase of the cell cycle. Kv1.5 controls myoblast proliferation through a mechanism involving the accumulation of the CDKIs p21^cip1^ and p27^kip1^ [91]. In oligodendrocyte progenitors, a similar increase in Kv1.3 and Kv1.5 expression is found at the G1 phase. However, Kv1.3 activity, rather than Kv1.5 activity, is involved in G1 progression in these cells. Similar to myoblasts, this mechanism involves the accumulation of CDKIs [92,93]. Many other examples of fluctuations in K^+^ channels expression and activity along the cell cycle are reported. For example, Kv1.2 and Kv2.1 mRNA are decreased from early to late G1, while K_Ca_3.1 increases in mesenchymal stem cells from the bone marrow. Such remodeling implies a decrease in voltage-gated delayed rectifier K^+^ currents and an increase in Ca-activated K^+^ currents throughout G1 progression. Knocking down each of these three genes impairs proliferation [94]. In spinal cord astrocytes, the downregulation of inwardly rectifying K^+^ currents is important for G1/S transition, whereas blockade of delayed outwardly rectifying currents causes G1 arrest. Conversely, a recovery of Kir currents is critical for mitosis. Furthermore, S-phase cell cycle arrest accumulates delayed outwardly rectifying currents [95]. 

K^+^ channels can also exhibit cell cycle-dependent localization. Kv2.1 is an intriguing example. This channel, which clusters at ER-PM junctions during mitosis, diffusely distributes during interphase. Such Kv2.1 transient localization is dependent on the phosphorylation state, which increases at M phase [49]. Kv2.1 stabilizes/enhances contacts between the endoplasmic reticulum and the plasma membrane, named as ER-PM junctions [96]. Therefore, the channel indirectly regulates localized Ca^2+^ movements and the composition of lipidic microenvironments, suggesting a structural role for Kv2.1 during mitosis. Kv10.1 is another example of cell cycle-dependent localization of K^+^ channels. This protein is located at the centrosome and primary cilium [97]. Disregarded for many years, the primary cilium is assembled at the plasma membrane of nearly all quiescent cells. Increasing evidence has pointed to the primary cilium as an important organelle for the transduction of extracellular information. However, the mechanism—either mechanical, chemical, or both—is still unclear. The primary cilium consists of a microtubule-based protrusion whose basal body derives from the mother centriole. Upon cell cycle entry, the primary cilium resorbs, and the centriole is redistributed to form the microtubule-organizing center (MTOC) [98]. Transient Kv10.1 expression is transcriptionally induced during G2/M transition by the direct binding of E2F1 to the Kv10.1 promoter [99]. The channel is then located at the basal primary cilium membrane where it promotes cilium resorption. The hypothesized mechanism postulates that local membrane hyperpolarization, due to Kv10.1 activity, would lead to increased Ca^2+^ influx and PIP2 dispersion from the basal cilium membrane; both events are necessary for primary cilium retraction [98]. Further examples of the importance of K^+^ channels localization for cell cycle regulation include Kv1.3. Inhibition of Kv1.3 activity at the plasma membrane blocks G1/S transition in many cell types. However, a recent study has shown that specific blockade of mitochondrial Kv1.3, with a low concentration of PAP-1 mitochondriotropic inhibitors, slightly favors proliferation, most likely by a mechanism involving mitochondrial ROS production [30].

The regulation of cell volume is intrinsically linked to changes in the membrane potential, which is crucial for cell cycle progression. Hyperpolarization via K^+^ channels activation favors Cl^−^ exits by increasing its electrical driving force. The consequent leakage of KCl implies cell shrinkage by osmotic water loss that, in turn, favors the initiation of Ca^2+^ oscillations driving cell proliferation. Moreover, water fluxes can modify the crowding of nutrients and other intracellular solutes, such as enzymes and co-factors involved in cell cycle regulation [100]. For instance, Kv11.1 peaks the expression at the G1 phase and directly connects with the cell volume regulation during the cell cycle. Sustained inhibition of channel activity causes cell bursting, which can be counteracted when decreasing intracellular osmotic pressure [101]. Interestingly, transient swelling, required for cell division, produces normal-sized daughter cells and regulates cell shape and cell-cell contacts [102].

In addition to flux-dependent abilities of K^+^ channels, the cell cycle can also be regulated by nonconducting properties of the channels. As transmembrane proteins, K^+^ channels can contribute to the initiation of many biochemical events by direct protein-protein interactions, leading to the initiation of many intracellular pathways. For instance, depolarization activates Kv1.3 by inducing a conformational change on its voltage sensor domain. This structural switch into the open state of Kv1.3 is sufficient to induce the channel pro-proliferative activity, independently of K^+^ conduction. Thus, a pore-less Kv1.3 promotes proliferation only if the voltage-dependence of gating is conserved [103]. Activation of Kv1.3 exposes a C-terminal docking domain, which contains different phosphosites essential to induce proliferation [104]. Similarly, Kv10.1 induces proliferation through the activation of the mitogen-activated protein kinase (MAPK) cascade in the absence of conducting properties [105]. These observations corroborate the importance of voltage-sensing flux-independent properties of K^+^ channels in the regulation of proliferation, which include conformational changes and the consequent gating currents.

## 5. Concluding Remarks

In recent years, countless examples of aberrant expression of Kv channels in several types of cancer have been described (Table 1). Their implication in tumor progression is variable. Thus, neoplastic transformation, proliferation, migration, adhesion, cell volume, or apoptosis—among other properties—can be altered when these proteins are remodeled.

Cell cycle regulation is related to the polarization state of the cells. As far as we know, changes in K^+^ channels expression or function may be a cause and/or consequence of changes in the membrane potential. Interestingly, highly proliferative cells show a depolarized phenotype. Inhibition of Kv has been related to cell cycle arrest and impairment of proliferation. On the other hand, Kv channels exhibit a cell cycle-dependent localization, which can be altered with different tumorigenic scenarios. Though the role of Kv channels in proliferation is highly demonstrated, their relationship to tumor progression is not entirely understood. The wide variety of tissues, cells, tumor stages, degrees of malignancy, and channels with related auxiliary proteins involved that can be affected further complicate our knowledge. Kv channels are, thus, seducing and exciting targets for anti-tumoral treatments. Toxins and blockers, acting selectively against Kv channels, impair tumor progression in vitro, although their use in vivo still deserves further work before use in human anticancer therapies.

## Figures and Tables

**Table 1:** Voltage-gated potassium channels and cancer.

Channel	Tissue	Modulation	Highlights	References
Kv1.3	Blood	↓	MitoKv1.3 is downstream of a pro-apoptotic signaling pathway. Kv1.3 inhibition promotes cell survival. Considered a tumor suppressor.	[25,26,27]
		↑	Upregulation of Kv1.3 in B lymphocytes is related to B-RAF signaling. Kv1.3 membrane-permeable inhibitors (clofazimine) induce apoptosis of B-CLL cells in the presence of mesenchymal stromal cells (anti-apoptotic).	[19,106]
		↓	No relation with tumor malignancy. Tumor suppressor. Role in apoptosis.	[19,26,28]
	Colon	↓	LS174 colon adenocarcinoma cell line. Methylation of the Kv1.3 promoter.	[38]
		↑	Kv1.3 modulates cell migration and adhesion but not apoptosis and proliferation.	[20]
	Brain	↓	Kv1.3 is downregulated in the plasma membrane of glioblastoma cell lines. MitoKv1.3-directed membrane-permeable drugs induce apoptosis in cell lines.	[31]
		↑	U87 Glioblastoma cell line. Kv1.3 modulates cell migration and adhesion but not apoptosis and proliferation.	[20]
	Breast	↑	MDA-MB-231 breast cancer cell line. Kv1.3 modulates cell migration and adhesion but not apoptosis and proliferation.	[20]
		↑	Breast cancer and tumorigenic human mammary epithelial cells. Kv blockers suppress tumorigenic cell proliferation.	[107]
		↓	Breast carcinoma samples and the MCF-7 cell line. Methylation of the Kv1.3 promoter increases in grade III tumors and cells. Related to poorly differentiated tumors and young patients.	[108]
	Prostate	↑↓	Protein levels vary from high to low expression in different primary prostate cancer patients. Low channel expression may correlate with the increased probability of metastatic disease.	[109,110]
	Pancreas	↑	Mito Kv1.3. Very aggressive and highly metastatic tumor. Correlated with high levels of anti-apoptotic Bcl-xL.	[30]
		↓	Methylation of the gene promoter.	[111]
		↓	Decreased expression in ductal adenocarcinoma grade II.	[25]
	Bones	↑	Osteosarcoma samples and derived cell lines.	[29]
	Skeletal muscle	↑	Increased expression in skeletal muscle carcinogenesis but no clear relationship with malignancy.	[35]
	Parathyroid	↑	DNA and protein overexpression of Kv1.3. Potential marker to distinguish carcinoma or adenoma.	[112]
Kv1.5	Blood	↓	Inversely correlates with aggressiveness in non-Hodgkin’s lymphomas.	[24]
	Skeletal muscle	↑	Increased expression in skeletal muscle carcinogenesis. Correlation with the degree of malignancy.	[35]
	Breast	↓	Absent or low expression in mammary duct carcinoma samples.	[25]
	Brain	↓	Kv1.5 inversely correlates with glioma malignancy. High in astrocytoma, moderate in oligodendroglioma, and low in glioblastoma.	[37]
	Skin	↑	High expression in squamous skin cell carcinoma.	[25]
	Colon	↑	Overexpression in colon adenocarcinoma.	[25]
	Stomach	↑	Kv1.5 may be involved in tumor cell proliferation by controlling calcium entry.	[33]
	Bone	↓	Promoters of ion channels are highly methylated in Ewing Sarcoma. Inhibiting CpG islands, cancer cells are sensitive to death. Kv1.5 would act as a tumor suppressor.	[39,40]
		↑	Osteosarcoma samples and cell lines. Silencing Kv1.5 impairs osteosarcoma cell proliferation and induces cell cycle arrest (G0/G1) and apoptosis.	[34]
Kv1.1	Breast	↑	Implicated in MDA-MB-231 breast cancer cell line migration and tumorigenesis via EGFR.	[20]
		↓	Tumor suppressor in primary mammary epithelia cancer samples and cell lines. Delocalization of Kv1.1 affects cellular senescence and transformation processes.	[46]
Kv2.1	Gastric	↑	Several gastric cancer cell lines.	[33]
	Cerebellum	↓	Medulloblastoma samples. Tumor suppressor. Heme Oxygenase-1 affects apoptosis via CO-mediated Inhibition of Kv2.1. Tumor cells become resistant to apoptosis.	[47]
	Cervix (uterus)	↑	Kv2.1/Kv9.3 participates in cell cycle regulation in cervical adenocarcinoma cells.	[48]
Kv7.1	Germinal	↑	High levels of KCNQ1/KCNE1 in human seminoma samples, characterized by the proliferation of undifferentiated germ cells.	[52]
	Colon	↑	Upregulated in human colorectal cancer and cell lines. Involved in TXA2-induced cancer cell proliferation.	[51]
Kv10.1	Brain	↑	Overexpression in primary brain tumor and metastases correlates with a poor prognosis. Antidepressants blocking Kv10.1 improve the survival rate in patients with moderate Kv10.1 expression.	[21]
		↑	Cell cycle-dependent expression in neuroblastoma cells.	[56]
	Colon	↑	Malignant colorectal adenocarcinomas. Enhanced function in carcinogenesis.	[59]
	Gastric	↑	Aberrant expression in gastric cancer tissues and cell lines. Role in proliferation in association with lymph node metastasis and cancer stage.	[60]
	Breast	↑	Kv10.1 expression induces cancer progression in several human cancer cell lines.	[54,57]
		↑	Correlation with the overexpression of HIF-1α in invasive ductal carcinoma samples. Close correlation with the clinical parameters of tumors. Interference with hypoxia homeostasis of the early stage of tumor progression.	[58]
	Bone	↑	Kv10.1 silencing inhibited cancer cell proliferation and colony formation via G1 phase arrest in the MG-63 osteosarcoma cell line.	[61,62]
Kv3.4	Lung	↑	Cell density- and hypoxia-dependent overexpression in A549 lung adenocarcinoma cell lines. Migration and invasion are affected in aggressive tumors.	[69]
	Oral, head and neck	↑	Leukoplakia and oral squamous cell carcinoma samples. Role in tumorigenesis, malignant transformation migration, and invasion.	[68,70]
Kv4.1	Gastric	↑	MKN-45 and SNU-638 gastric cancer cell lines. Inhibition of Kv4.1 impairs cell proliferation and cell cycle distribution.	[72]
	Breast	↑	M13SV1 mammary epithelial cells and breast cancer samples. Kv4.1 positively correlates with malignant stages.	[73]
Kv9.3	Cervix (uterus)	↑	Kv2.1/Kv9.3 participates in cell cycle regulation in cervical adenocarcinoma cells.	[48]
	Colon	↑	Kv2.1-independent role in cancer progression. Kv9.3 blockade halts tumor cell proliferation by arresting the cell cycle at G0/G1.	[75]
	Lung	↑	Kv2.1-independent role in cancer progression. Kv9.3 blockade halts tumor cell proliferation by arresting the cell cycle at G0/G1.	[75]
Kv11.1	Gastric	↑	Crucial in the P13K/Akt-dependent pathway that induces HIF and VEGF to promote tumor progression. Blocking Kv11.1 inhibits cell growth, angiogenesis and metastasis.	[22]
	Pancreas	↑	High levels in primary PDAC samples and related to EGFR. Channel blockade impairs PDAC cell line growth and migration.	[80]
	Breast	↑	Present in all breast cancers. Kv11.1 is associated with a better prognosis and lower metastasis rate.	[82]
